# Genome-wide incorporation dynamics reveal distinct categories of turnover for the histone variant H3.3

**DOI:** 10.1186/gb-2013-14-10-r121

**Published:** 2013-10-31

**Authors:** Daniel C Kraushaar, Wenfei Jin, Alika Maunakea, Brian Abraham, Misook Ha, Keji Zhao

**Affiliations:** 1 Systems Biology Center, National Heart, Lung, and Blood Institute, NIH, Bethesda, MD, 20892, USA; 2 Samsung Advanced Institute of Technology, Samsung Electronics Corporation, Yongin-Si, Gyeonggi-Do 446-712, South Korea

## Abstract

**Background:**

Nucleosomes are present throughout the genome and must be dynamically regulated to accommodate binding of transcription factors and RNA polymerase machineries by various mechanisms. Despite the development of protocols and techniques that have enabled us to map nucleosome occupancy genome-wide, the dynamic properties of nucleosomes remain poorly understood, particularly in mammalian cells. The histone variant H3.3 is incorporated into chromatin independently of DNA replication and requires displacement of existing nucleosomes for its deposition. Here, we measure H3.3 turnover at high resolution in the mammalian genome in order to present a genome-wide characterization of replication-independent H3.3-nucleosome dynamics.

**Results:**

We developed a system to study the DNA replication-independent turnover of nucleosomes containing the histone variant H3.3 in mammalian cells. By measuring the genome-wide incorporation of H3.3 at different time points following epitope-tagged H3.3 expression, we find three categories of H3.3-nucleosome turnover *in vivo*: rapid turnover, intermediate turnover and, specifically at telomeres, slow turnover. Our data indicate that H3.3-containing nucleosomes at enhancers and promoters undergo rapid turnover that is associated with active histone modification marks including H3K4me1, H3K4me3, H3K9ac, H3K27ac and the histone variant H2A.Z. The rate of turnover is negatively correlated with H3K27me3 at regulatory regions and with H3K36me3 at gene bodies.

**Conclusions:**

We have established a reliable approach to measure turnover rates of H3.3-containing nucleosomes on a genome-wide level in mammalian cells. Our results suggest that distinct mechanisms control the dynamics of H3.3 incorporation at functionally different genomic regions.

## Background

Incorporation of histone variants into chromatin critically influences the properties of nucleosomes that play important roles in regulating transcription and epigenetic memories [1-6]. The histone variant H3.3 differs from canonical H3 by a few amino acids and is ubiquitously expressed in eukaryotes. Different from canonical histone H3, which is expressed in S phase and is incorporated into chromatin during DNA replication, H3.3 can be incorporated into chromatin independent of DNA replication [7,8]. The incorporation of histone variants is tightly regulated by histone chaperones. The H3.3-specific chaperones Atrx-Daxx and HIRA deposit H3.3 primarily at telomeres and non-heterochromatic regions, respectively [9-12]. Despite small differences in amino acid sequence between H3.1, H3.2 and H3.3, each is distributed differentially across the genome and carries its own characteristic histone modification signature, strongly suggesting distinct functional roles for each H3 variant [9]. Histone marks that are associated with gene activation, such as acetylation marks and H3K4me3, are typically found on H3.3 whereas H3K27me2 and H3K9me3 are found on H3.2 [13]. Marks associated with gene silencing are predominantly found on H3.1. However, the precise relationship between H3.3 deposition and transcription are not well understood. In *Drosophila*, H3.3 replaces H3 during gene activation and becomes deposited in active chromatin and especially very highly expressed ribosomal DNA [14,15]. In mammalian tissues the pattern of H3.3 enrichment is also associated with gene activity and H3.3 is generally associated with the transcription start site (TSS), transcription end site (TES) and gene bodies of active genes [16], although the unique chromatin of embryonic stem cells (ESCs) also carries H3.3 at promoters of certain inactive genes [1,9]. In spite of their independent evolution, plant H3.3 and H3.1 display a broadly similar distribution to animal H3 variants, which indicates a conserved function for H3 variants [17]. Nucleosome occupancy and positioning are critical to regulating gene transcription and epigenome maintenance [18,19]. In mammalian cells, although nucleosomes are present at promoters and enhancers, they must be dynamically regulated to accommodate binding of transcription factors and RNA polymerase machineries by various mechanisms [20-23].

Intrinsic nucleosome characteristics such as inclusion of histone variants as well as extrinsic factors such as ATP-dependent nucleosome remodeling, the transcriptional machinery and various other factors shape the dynamic profile of nucleosomes [24,25]. Despite the development of protocols and techniques that have enabled us to map the genome-wide nucleosome occupancy, their dynamic properties are only poorly understood. Our current understanding of the dynamics of nucleosomes comes from studies performed in yeast and *Drosophila*. Transgenic epitope-tagged histones can be inducibly expressed to estimate nucleosome turnover and allow detection of specific histone incorporation [26-28]. Alternatively, newly synthesized, native histones can be metabolically labeled with an amino acid analogue that is coupled to an affinity tag (CATCH-IT), which allows for detection of H3/H4 tetramers [29]. Using these strategies, it has been shown that nucleosome exchange is rapid at promoters and coding regions, and relatively slower at heterochromatic regions. Furthermore, differential turnover can be quite localized. For instance, faster nucleosome turnover has been detected at Trithorax-group binding sites than at polycomb group protein binding sites [26,30].

Apart from the measurement of turnover, inducible expression systems with tagged histones have also contributed to our understanding of mechanistic aspects that pertain to histone deposition. For instance, studies from yeast have shown that Asf1 is required for the deposition of H3 and that the amino termini of both H2B and H3 are not required for their incorporation into nucleosomes [27,28].

While these techniques measure average histone deposition rates across cell populations, alternative techniques such as FRAP and SNAP-tag have allowed the deposition of histones in individual cells to be visualized [31-33].

The genome-wide turnover of the histone variant H3.3 in mammals has not been studied up until now. Global FRAP studies in HeLa cells with green fluorescent protein (GFP)-tagged histones revealed only cycling of H2B. Canonical H3 and H4, in contrast, exhibited very slow cycling, and the majority of H3 remained permanently bound outside S phase [33]. Slow turnover of core histones may be a feature of somatic cells since core histone exchange is substantially more rapid in pluripotent ESCs than in differentiated cell types [34]. Hence, fast turnover may be inherently linked to cell plasticity.

In this study, we developed a versatile technique to map the dynamics of histone variant incorporation into chromatin in mammalian cells. Using this technique, we mapped the replication-independent incorporation of the histone variant H3.3 in mouse embryonic fibroblasts (MEFs). We were able to track H3.3 incorporation across a relatively short time window of several hours after induction of H3.3 as well as over a longer time frame of up to 72 hours. By combining our chromatin immunoprecipitation (ChIP)-based technique with high-throughput sequencing, we measured the H3.3-nucleosome turnover kinetics at the genome-wide level. Our results reveal three major categories of H3.3 nucleosome turnover: (1) rapid turnover at enhancers and promoters; (2) intermediate turnover at gene bodies; and (3) slow turnover at heterochromatic regions. We find faster H3.3 turnover at enhancers and promoters is positively correlated with active histone modifications, including H3K4me1, H3K4me3, H3K9ac, H3K27ac and the histone variant H2A.Z, whereas slower turnover is negatively correlated with H3K27me3 and H3K36me3 modifications. These results show that distinct mechanisms of histone deposition and eviction pertain to the dynamics of nucleosomes at different functional chromatin regions. We also show that turnover is related to the presence of specific histone marks, strongly suggesting that histone modifications are important determinants of nucleosome stability.

## Results

### An ectopic expression system to measure turnover of H3.3

**Figure 1:**
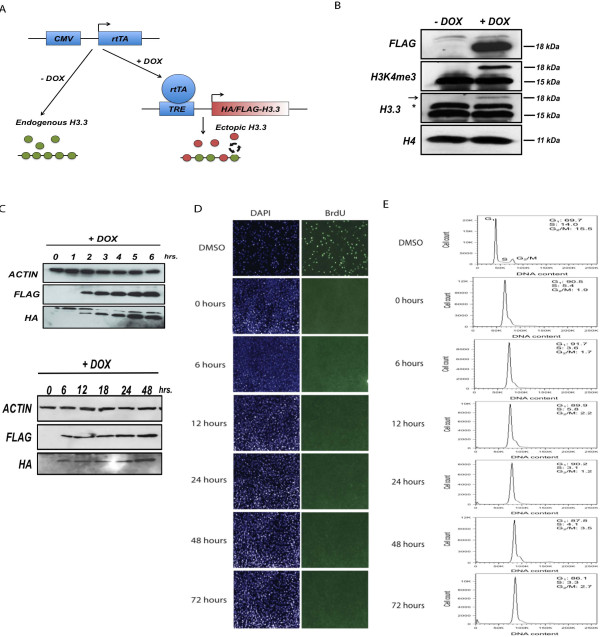
**A versatile system to study replication-independent nucleosome dynamics in mammals. (A)** Schematic of TET-inducible expression system to study H3.3 turnover. CMV, cytomegalovirus; rtTA, reverse tetracycline-controlled transactivator; TRE, tetracycline responsive elements. **(B)** Western blot showing protein levels of transgenic HA/FLAG-H3.3 compared to endogenous H3.3. HA/FLAG-H3.3 expression 24 hours after DOX addition. The band marked with an asterisk is non-specific. The arrow marks transgenic HA/FLAG-H3.3. **(C)** Time course western blots of HA/FLAG-H3.3 expression. **(D)** Bromodeoxyuridine (BrdU) immunostaining of NIH/3 T3 cells treated with DNA polymerase inhibitor aphidicolin and DOX across time points of H3.3 induction. DMSO, dimethylsulfoxide. **(E)** Cell cycle analysis of cells treated with aphidicolin/DOX. Cells were stained with propidium iodide and analyzed by flow cytometry.

In order to track histone incorporation and thereby assay the genome-wide dynamics of the histone variant H3.3, we generated MEFs that carry a cytomegalovirus-controlled tetracycline (TET) transactivator and hemagglutinin (HA)/FLAG-tagged H3.3 expression cassette controlled by tetracycline response elements. This TET-ON expression system allowed us to induce the expression of a HA/FLAG-tagged version of H3.3 by addition of the tetracycline analog doxycycline (DOX) (Figure 1A). In our tetracycline inducible-HA/FLAG-H3.3 MEF cell line, we detected robust H3.3 expression as early as 2 hours after DOX addition that continued to increase until 48 hours after DOX addition. No tagged H3.3 expression was detected in the absence of DOX (Figure 1B). Immunoblotting against H3.3 revealed that transgenic H3.3 expression levels were only a small fraction of those of endogenous H3.3. Furthermore we verified that the HA/FLAG tags did not interfere with the H3K4me3 modification of H3.3 (Figure 1C).

In order to minimize the effect of replication-coupled histone disassembly, we arrested the cell cycle of confluent NIH/3 T3 MEF cells by treatment with the DNA polymerase (Pol) inhibitor aphidicolin. After 18 hours of aphidicolin treatment (time point 0) and across the time course of HA/FLAG-H3.3 induction, the MEF cell population was essentially devoid of cells in S phase and arrested at the G1/S phase boundary, as indicated by bromodeoxyuridine and propidium iodide staining (Figure 1D,E). Thus, to monitor the genome-wide dynamics of replication-independent H3.3 incorporation, we induced HA/FLAG-H3.3 expression in cells arrested by aphidicolin, followed by ChIP-Seq analysis using the HA antibody at various time points. We took a ‘high-resolution’ approach by tracking histone incorporation across hourly time points of early protein expression and across a longer time frame of up to 48 hours.

### Genome-wide characterization of H3.3 incorporation

**Figure 2:**
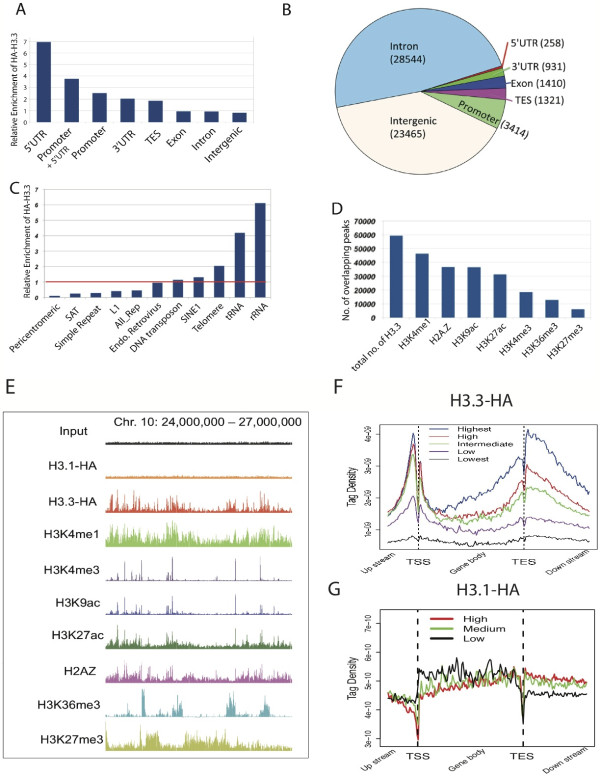
**Distribution and enrichment of H3.3 in the genome. (A)** Enrichment of HA-H3.3 at various genomic regions of different annotation. The total read count of each category was normalized over its total length. **(B)** Pie chart showing the proportions and numbers of peak centers that fall into various genomic categories. **(C)** Enrichment of HA-H3.3 at various repetitive elements. All_Rep, all repetitive elements; SAT, satellite regions; SINE, short interspersed nuclear element. **(D)** Count of histone mark peaks that overlap with HA-H3.3 peaks. Overlapped peaks were defined as overlapping when one peak (H3.3 or histone mark) shared 10% of reads with the other. ChIP-Seq libraries for various histone marks were prepared from cells treated with aphidicolin. **(E)** UCSC Genome browser view illustrating overlap of HA-H3.3 with various histone marks and H2AZ. **(F)** Distribution profile of HA-H3.3 based on mRNA expression levels as indicated by different colors. RNA-seq libraries were prepared from cells treated with aphidicolin. **(G)** Distribution profile of HA-H3.1 based on mRNA expression levels as indicated by different colors. ChIP-Seq and RNA-Seq libraries were prepared from untreated (no aphidicolin) cells.

In order to characterize the genome-wide deposition of HA-H3.3, we sought to map the H3.3 distribution 72 hours post-induction. Consistent with previous reports from HeLa and mouse ESCs [1,9], we found that H3.3 is strongly enriched in promoters, 5’ UTRs, as well as the 3’ ends of genes (Figure 2A,B,F). In terms of read density, promoters and TES regions were overrepresented in the H3.3-enriched genomic regions (Figure 2A). The majority of H3.3 peaks were detected at introns followed by peaks in intergenic regions (Figure 2B). In line with the idea that H3.3 carries primarily active histone modifications [13], we found strong overlap of H3.3 with the distribution profiles of histone modifications such as H3K4me1, H3K4me3, H3K9ac and H3K27ac. In contrast we detected substantially fewer regions of H3.3 incorporation at sites enriched with H3K27me3 (Figure 2D, 2E). Since the vast majority of sharp H3.3 peaks in introns and intergenic regions were associated with enhancer marks, including H3K4me1, H3K27ac and H2A.Z, we considered them as putative enhancers in the following analyses.

Although most of the repetitive elements showed obvious depletion of H3.3, high levels of H3.3 were detected at telomeres, rRNA and tRNA repeats (Figure 2C). H3.3 co-localizes with pericentromeres in several differentiated cell types [10]. We, however, did not find significant deposition of HA-H3.3 at pericentromeric regions. This strongly suggests that H3.3 deposition in these regions is a strictly replication-coupled process as we profiled the distribution of HA-H3.3 from cell cycle arrested and non-dividing cells.

To test the relationship between H3.3 incorporation and gene expression, we plotted the average profiles of H3.3 across several groups of genes sorted according to their expression levels (Figure 2F). Unlike in mouse ESCs, we found that H3.3 is associated mostly with active genes and not inactive genes.

Enrichment levels of H3.3 at TSSs, gene bodies and TESs were positively correlated with the transcription level of the associated genes (Figure 2F). Interestingly, we find that the relation between HA-H3.3 enrichment and gene expression follows a bimodal distribution at promoters. Only promoters of expressed genes carry substantial amounts of H3.3, and H3.3 enrichment levels do not increase further at even higher expression levels. In contrast, HA-H3.3 enrichment in gene body and TES regions was strongly positively correlated with gene expression levels. This indicates that H3.3 in gene bodies may be directly transcription coupled, whereas other mechanisms of H3.3 deposition may exist upstream of TSSs. As a control alongside H3.3, we generated a MEF cell line that expressed canonical HA-H3.1 instead of HA-H3.3, which differs in five amino acids from H3.3, and mapped its replication-coupled deposition. Changing H3.3 to H3.1 abolished the defined enrichment of H3.3 to a homogenous distribution similar to reads mapped from input samples, confirming its universal deposition throughout the mammalian genome (Figure 2E). Furthermore, the pronounced pattern of H3.3 enrichment around the TSS, gene body and TES was lost. Slightly higher levels of H3.1 were detected around the TSSs of inactive and low-expressed promoters, indicating prevalence of H3.1 at inactive genes (Figure 2G). Similar genome-wide ChIP-Seq profiles were obtained by Goldberg *et al.*[9] with zinc finger-targeted, heterozygously tagged H3.3B in ESCs: mutating HA-H3.3 to HA-H3.2 or to a HA-H3.1S31 hybrid altered the H3 distribution to a genome-wide pattern similar to that of input samples or ChIP-Seq profiles obtained with antibodies against general H3.

### H3.3 displays early and late chromatin incorporation

**Figure 3:**
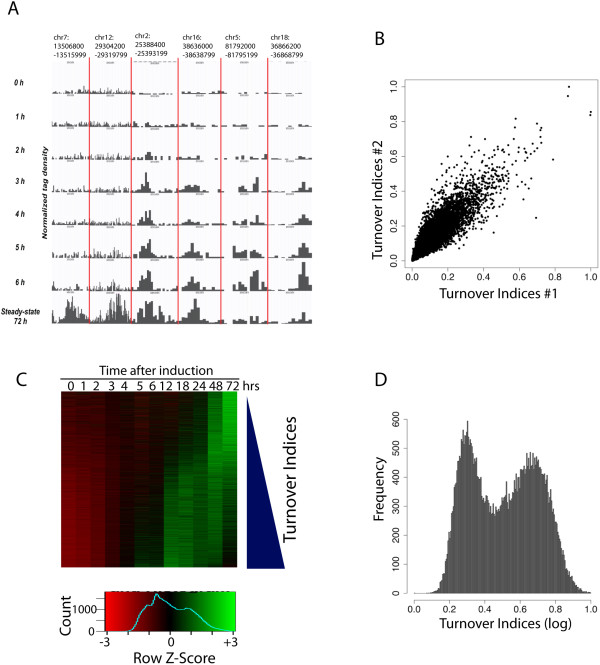
**H3.3 nucleosomes display fast and slow turnover rates. (A)** Genome browser view of HA-H3.3 profiles at several genomic regions over a 6-hour time frame. Examples of HA-H3.3 peaks that exhibit slow (left two panels), intermediate (center two panels) and fast turnover (right two panels) are displayed. **(B)** Dot plot of turnover indices calculated from two independent experiments. **(C)** Heat map showing relative HA-H3.3 enrichment (red = low, green = high) across all time points sorted by turnover index from low to high. **(D)** Distribution of turnover indices reveals two populations of H3.3 nucleosome exchange.

The above results indicated the steady-state enrichment of H3.3 across the genome. In order to measure turnover rates of H3.3, we profiled the dynamics of HA-H3.3 incorporation after 0, 1, 2, 3, 4, 5, 6, 12, 18, 24 and 48 hours of DOX treatment (Figure 3A). Using a linear regression model, we calculated a turnover index for each individual H3.3 peak that was identified using SICER and found that the turnover indices are highly reproducible from two independent experiments (Figure 3B).

We observed very different H3.3 incorporation kinetics across the genome. A substantial number of sharp peaks appeared within 2 to 3 hours of DOX induction and reached their maximum enrichment well before the end point of the induction (Figure 3A,C). The subsequent decline in read numbers that was observed at peaks with rapid H3.3 deposition may be a result of ‘read shifting’. As the total read coverage becomes saturated, more reads may shift to peaks, which appear at later stages of induction. Importantly, this way, sites of enrichment may be identified, which would otherwise go undetected in a steady state. Meanwhile, we also observed many broad peaks, mainly in transcribed gene body regions, which took 12 to 24 hours to be detected (Figure 3A,C). Plotting the distribution of turnover rates revealed the existence of two populations of H3.3 peaks with slow turnover and fast turnover, respectively (Figure 3D).

### Distinct H3.3 exchange rates in promoters, enhancers and gene bodies

**Figure 4:**
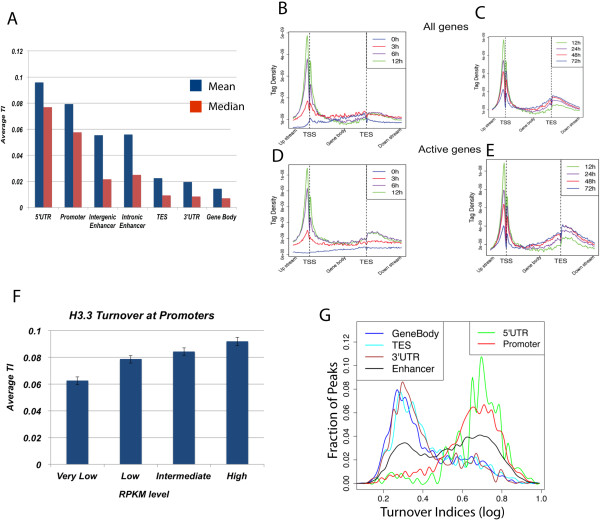
**Promoters and enhancers are associated with fast H3.3 nucleosome turnover whereas gene body and transcription end site regions are associated with slow H3.3 nucleosome turnover. (A)** Median and mean turnover indices (TIs) calculated for various genomic regions. Intergenic enhancers were defined as peaks that are located in intergenic regions at least 1 kb from the TSS. Intronic enhancers were defined as peaks located in introns only. Gene body regions were defined as broad peaks that spanned both intronic and exonic regions and shared significant overlap with H3K36me3. **(B-E)** Distribution profiles of HA-H3.3 from TSS to TES at different time points. **(F)** Distribution plot illustrating the range of turnover indices within various genomic categories. Turnover rates were log-normalized. RPKM, reads per kilobase of exon model per million reads. **(G)** Distribution plot illustrating the range of turnover indices of H3.3 nucleosomes within various genomic categories. Log of turnover indices was calculated and re-scaled from 0 to 1.

To examine the H3.3 turnover rates at different genomic regions, we calculated the average mean and median turnover indices at each region (Figure 4A). The analysis revealed that 5’ UTR and promoter regions generally have the highest turnover indices; enhancers have the second highest, and 3’ UTR and TES regions have the lowest turnover indices (Figure 4A). To examine the timing of H3.3 incorporation in these different regions, we compared the average profiles of H3.3 density of either all genes or active genes for the first 12 hours or after 12 hours till 72 hours of induction (Figure 4B-E). Notable incorporation of HA-H3.3 was detected at the promoter region within 3 hours. Further increases in deposition of HA-H3.3 were observed within 6 hours at the TSS and the majority of genes reached their maximum by 12 hours before starting to decline (Figure 4B,D). Remarkably, the onset of HA-H3.3 deposition at gene bodies was not detected any time before the 6-hour time point (Figure 4C,E). H3.3 deposition at gene bodies continued to increase from 12 hours to 72 hours of induction, when it reached the steady-state level. It is interesting to note that the signal of HA-H3.3 at the TSS exhibited decreases from 12 hours to 72 hours.

High turnover of H3.3 in promoter regions of active genes suggests that nucleosome turnover may be correlated with transcriptional activation. Further examination of turnover rates at promoters indeed showed a modest but positive correlation with gene expression levels (Figure 4F). The relationship between promoter turnover and gene transcription suggests additional mechanisms by which nucleosome exchange facilitates and/or is facilitated by gene activation.

To further examine variation of the H3.3 turnover rates within each genomic category, we plotted the distribution of turnover indices separated into these categories (Figure 4G). The analysis revealed a relatively narrow range for the high turnover at promoters and 5’ UTRs and slow turnover at gene bodies and 3’ UTRs, respectively, suggesting that the H3.3 nucleosome exchange in these regions are controlled by distinct mechanisms at these respective sites (Figure 4G). Interestingly, enhancer regions exhibited broad variability with regards to their turnover rates, indicating that not all regulatory regions are marked by high nucleosome turnover. Instead additional factors such as histone variants or histone modifications may contribute to nucleosome stability and turnover.

### Rapid H3.3 nucleosome turnover is associated with active histone marks at promoters and enhancers

**Figure 5:**
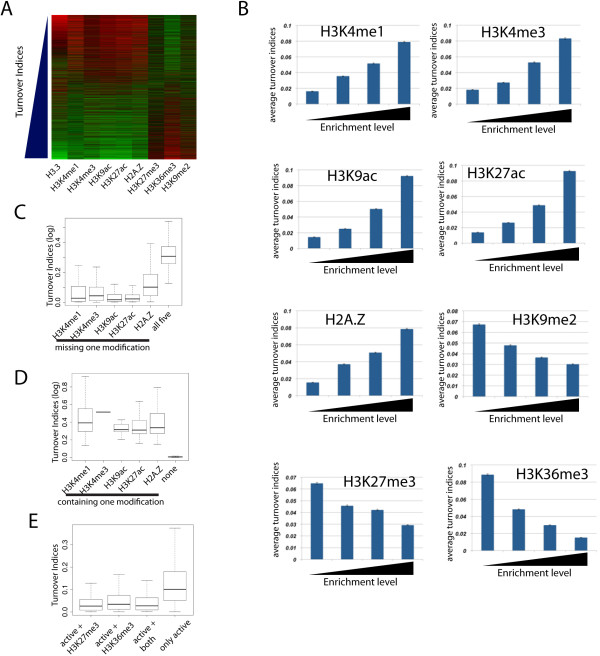
**Fast turnover rates are associated with active histone modifications. (A)** Heat map illustrating the relation between turnover rates and histone marks. Turnover indices were sorted from low to high (top to bottom). Relative enrichment (red = low, green = high) levels of various histone marks is shown. **(B)** Enrichment values for various histone marks were grouped into four bins with increasing enrichment levels and mean turnover indices were calculated for each bin. **(C)** Missing any of the five ‘active’ modifications decreases the H3.3 turnover index of enhancer nucleosomes. The turnover indices for enhancer H3.3 nucleosomes containing all five active modifications or missing one single modification are indicated on the y-axis. **(D)** Association with any of the five active modifications increases the turnover index of H3.3-nucleosomes at enhancers. The turnover indices for enhancer H3.3 nucleosomes containing none of the five active modifications or only one modification are indicated on the y-axis. **(E)** Association with either H3K27me3 or H3K36me3 decreases the turnover index of H3.3 nucleosomes. The turnover indices are indicated on the y-axis for H3.3 nucleosomes containing all five active modifications (only active) or five active modifications plus either H3K27me3 or H3K36me3 or both.

Several studies have shown that H3.3 is enriched in transcriptional regulatory regions such as promoters and enhancers [1,9,15]. Consistent with these observations, we found that many H3.3 peaks are co-localized with active histone modification marks, including H3K4me1, H3K4me3, H3K9ac, H3K27ac and the histone variant H2A.Z, that are often associated with promoters and enhancers (Figure 2D,E) [1,35,36]. To elucidate the relationship between H3.3 nucleosome turnover and histone modifications, we sorted all H3.3 peaks based on their turnover rates and displayed histone modification signals using heatmaps. The analysis indicated that faster turnover rates are generally associated with higher levels of H3K4me1, H3K4me3, H3K9ac, H3K27ac and H2A.Z, whereas slower turnover is associated with higher levels of H3K27me3 (Figure 5A). More quantitative analyses confirmed that indeed H3.3 peaks with higher levels of active modifications are turned over faster. In contrast, H3.3 peaks associated with heterochromatic marks such as H3K9me2 and repressive marks such as H3K27me3 are turned over slower (Figure 5B).

To understand the differences between the enhancers with fast H3.3 turnover and those with slow H3.3 turnover (Figure 4G, black line), we compared the histone modifications associated with these two groups of enhancers (Additional file 1). The analysis revealed that enhancers that displayed high turnover carried significantly higher median levels of active chromatin marks whereas enhancers of low turnover carried higher levels of H3K27me3 (Additional file 1). Unexpectedly, our data indicated a negative correlation between H3.3 turnover and H3K36me3 levels (Figure 5A,B), in line with the idea that H3K36me3 stabilizes nucleosomes in order to prevent cryptic transcription [37,38].

To evaluate the relative contribution of individual histone modifications to turnover rates, we compared the group of enhancers associated with all five active modifications to groups missing single histone marks. The results indicate that the turnover rates decreased significantly by missing any of the active modifications (Figure 5C). Consistent with this observation, association with only one of these modifications increased the turnover rates compared to the enhancers without these modifications (Figure 5D). In contrast to these active modifications, inclusion of either H3K27me3 or H3K36me3 substantially decreased the turnover index of H3.3 nucleosomes (Figure 5E).

In summary, we find that H3.3 exchange rate is positively correlated with active chromatin modifications (H3K4me1, H3K4me3, H3K9ac, H3K27ac and H2A.Z) and negatively correlated with repressive marks (H3K9me2 and H3K27me3). While the steady-state level of H3.3 is positively correlated with H3K36me3, its turnover rate is negatively correlated with H3K36me3.

### The kinetics of HA-H3.3 incorporation at tRNA and rRNAs genes

**Figure 6:**
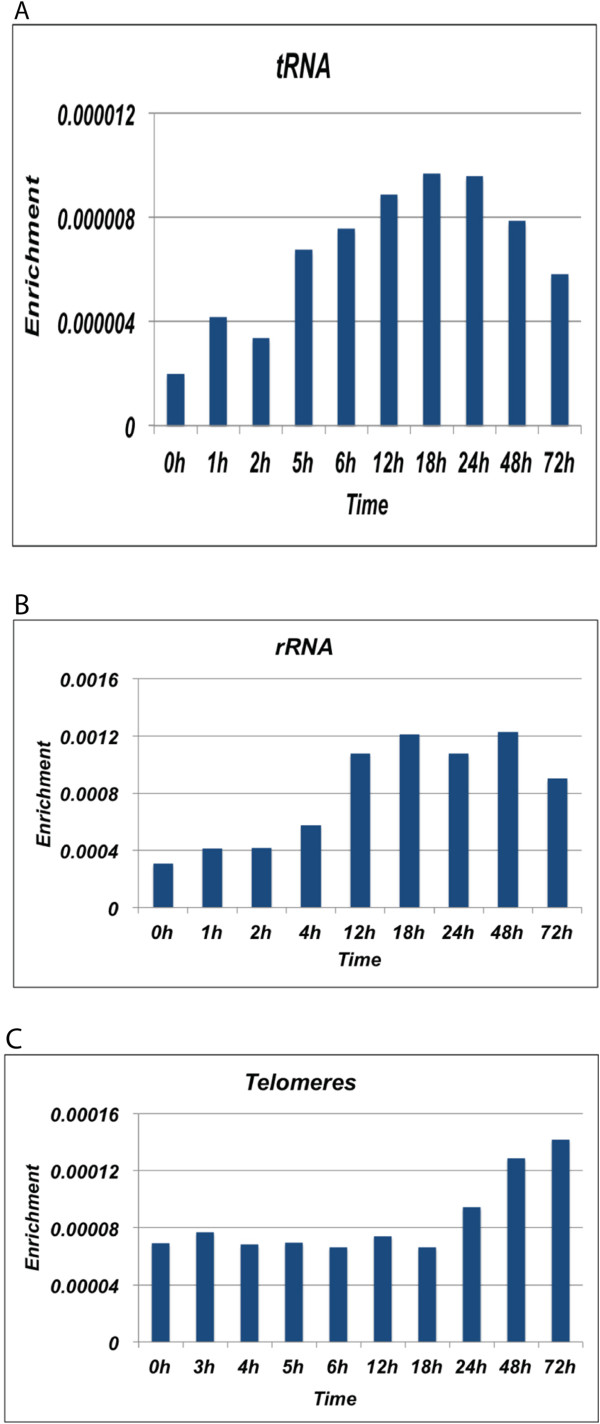
**Different repetitive sequences display distinct H3.3 turnover rates. (A-C)** Mapping of HA-H3.3 to repetitive elements across all time points showing turnover of H3.3 at tRNA **(A)**, rRNA **(B)** and telomeric repeats **(C)**.

We found that HA-H3.3 is enriched in tRNAs and rRNA genes (Figure 2C). The incorporation of HA-H3.3 was apparent from 6 hours of induction and continued to increase till 24 hours before it started to decline at 48 hours (Figure 6A). The incorporation of HA-H3.3 at rRNA genes was apparent at 12 hours and reached its maximum at 48 hours (Figure 6B). Both tRNA and rRNA exist as tandem repeat regions, are highly expressed and are transcribed by Pol III and Pol I, respectively. Pol III-transcribed genes share many epigenetic characteristics with Pol II transcribed genes, such as presence of H3K4me1/2/3 and absence of H3K27me3 [39]. Hence, high turnover at tRNA and rRNA repeats is consistent with our previous result that demonstrated high H3.3-nucleosome turnover at sites marked with active histone marks. High nucleosome exchange may directly facilitate accessibility of Pol III-specific transcription factors TFIIIB and TFIIIC to provide functional promoters and assist in transcriptional initiation.

### Slow H3.3 replacement in telomeres

Compared to promoters, enhancers, gene body regions, tRNAs and rRNAs, the incorporation of HA-H3.3 at telomeres exhibited the slowest rate. It was detected at 48 hours and reached a maximum at 72 hours after induction (Figure 6C), suggesting that a different mechanism for the HA-H3.3 deposition may take effect at telomeres. Heterochromatic regions are expected to have stable nucleosomes and most of their histone exchange is expected to take place during S phase. Yet, we observed slow rates of HA-H3.3 replacement at telomeres outside S phase, indicating slow replication-independent histone exchange that may be necessary for maintenance of telomeric stability. It is known that repetitive features of telomeres and pericentromeres have a relatively low affinity for nucleosomes and this may contribute to histone displacement [40].

## Discussion

Nucleosome dynamics and histone turnover are not well understood in mammals. In this report, we have developed a robust, TET-inducible system to study the kinetics of genome-wide deposition of the histone variant H3.3 in MEF cells. Using this system, we were able to measure turnover rates of genome-wide H3.3 target sites and were able to infer important differences between turnover rates of various genomic regions. Several categories of H3.3 deposition kinetics were observed: invariably high turnover at promoters, a broad range of turnover rates at enhancers, and slow turnover at gene bodies. In addition, repeat elements displayed dramatically different turnover rates with relatively high turnover at tRNA and rRNA elements and very slow H3.3 exchange at telomeres. These results provide novel insights into the genome-wide turnover of H3.3-containing nucleosomes and suggest distinct mechanisms of nucleosome assembly, stability and eviction in order to fulfill their function in regulating transcription and maintaining chromatin stability and integrity.

### Genome-wide incorporation of H3.3

Unlike canonical histones, the histone variant H3.3 can be deposited onto chromatin in a replication-independent manner [7,8]. It is enriched in transcriptionally active regions, such as gene bodies and promoters and enhancers of mammalian cells [1,9]. Incorporation of H3.3 into chromatin destabilizes the nucleosome structure and may facilitate transcriptional activation by generating a more accessible chromatin configuration [1,2]. Its deposition at promoters as well as in gene bodies is associated with epigenetic inheritance and may contribute to epigenetic memories [3,16].

Our analysis of H3.3 distribution at the steady state revealed its presence at intergenic regions that overlap with histone modifications that typically mark enhancers, such has H3K4me1, H3K27ac and H2A.Z, in addition to the enrichment at TSS regions. In coding regions, we find that H3.3 is broadly distributed within the gene body of actively transcribed genes as well as TESs and regions immediately after. This association is well correlated with gene expression levels as has been reported from other cell lines [1,9]. At promoter regions we find strong differences in H3.3 enrichment between inactive genes and active genes but relatively moderate differences between different expression groups, which follows an almost bimodal distribution. Based on H3.3 incorporation studies that followed H3.3 incorporation over time it was suggested that H3.3 incorporation at promoters was constitutive as only minor increases in H3.3 enrichment were observed upon interferon stimulation [16]. Our data argue against a direct transcription-coupled mechanism of incorporation at promoters as expression levels and enrichment levels are bimodal, which further suggests that the H3.3 incorporation may function upstream of the transcriptional activation. Enrichment of H3.3 within gene body regions and at the TES showed strong correlations with transcription levels, in line with the notion that H3.3 incorporation in gene bodies is directly coupled to elongation. The ChIP-Seq profiles between gene body H3.3 and Pol II are highly similar. Furthermore, a physical interaction between HIRA and Pol II has been demonstrated and provides further testament for a direct link between H3.3 deposition and transcriptional elongation [32]. The role of H3.3 deposition in gene bodies remains unclear but may be implicated in conferring epigenetic inheritance in order to facilitate constitutive transcription of genes after cell replication has taken place.

### Dynamics of H3.3 incorporation and differential turnover

Our results on the dynamics of H3.3 incorporation revealed three basic modes of H3.3 deposition kinetics. The most dynamic exchange of H3.3 was observed at promoters and enhancers. Robust signals of induced H3.3 were detected within 2 to 3 hours of induction at these regulatory regions, which is consistent with the rapid nucleosome turnover at chromatin boundaries observed in yeast [26]. The second category of intermediate-rate incorporation of newly induced H3.3 was found at gene body regions of active genes. The incorporation of H3.3 to gene bodies became apparent at 12 hours and reached a maximum at 72 hours. The slowest incorporation of H3.3 was detected at telomeres, with detectable H3.3 deposition at around 24 to 48 hours post-induction. Such differential turnover suggests that multiple mechanisms of H3.3 deposition and displacement occur, which no doubt will become subject to further investigation.

The fast turnover at enhancers and promoters may involve histone chaperones such as HIRA and Atrx-Daxx and other chromatin remodeling enzymes as well as sequence-specific transcription factors. High turnover at promoters suggests that high nucleosome turnover is a pre-requisite for binding of the transcriptional machinery as well as transcription factors.

The concept of nucleosome versus transcription factor competition has been well established from the Pho5 promoter in yeast [19,23,41]. Without stimulation, nucleosomes are bound to the binding sites of the transactivator Pho4 and effectively repress expression of the gene. Only upon release of nucleosomes at the promoter can Pho4 bind to its target site and gene activation effectively occur [23]. Unlike in yeast, active promoters of mammalian cells are occupied by nucleosomes [20]. So what is the role of their turnover? It is attractive to speculate that nucleosomes sterically compete with DNA binding factors and that accessibility can only be granted when nucleosome turnover is high [26].

Although simple nucleosome depletion could accomplish accessibility, ‘transient’ nucleosome deposition may stabilize DNA integrity, which may be necessary for accurate transcription factor binding. Several families of transcription factors, so-called ‘pioneer factors’ [42], require the presence and ‘guidance’ of nucleosomes for proper binding and subsequent nucleosome remodeling [43]. Secondly, nucleosomes and their histone modifications provide docking stations for a variety of transcription factors and chromatin remodeling factors, which may require continuous recruitment for initiation of transcription. Continuous nucleosome turnover may allow for accessibility and nucleosome binding for chromatin factors at the same time, which could be facilitated by the presence of multiple active modifications at enhancers or promoters. In contrast, repression of transcription and epigenetic inheritance may require greater stability of nucleosome organization and thus slower turnover at these sites, which, in turn, could be facilitated by association with polycomb group proteins and H3K27me3 modification [30].

The modest rate of H3.3 incorporation in gene bodies and TES regions is closely related to active transcription, and the accumulative signals of H3.3 in these regions may require multiple rounds of transcription of a gene unit. It is generally thought that RNA polymerase displaces nucleosomes during elongation, as it cannot pass by nucleosomes ahead of it [44,45]. Nucleosomes must be replaced behind passing RNA polymerase in order to maintain the fidelity of transcription and avoid cryptic transcription [46]. Unlike other histone chaperones, HIRA possesses unique DNA binding properties that allow it to bind to naked DNA. Due to its physical association with Pol II, a ‘gap filling’ mechanism has been proposed by which HIRA under the guidance of PolII will fill protective H3.3-nucleosomes into spaces of naked DNA [32]. Our study reaffirms the continuous eviction and replacement of H3.3-containing nucleosomes during transcription. Slow exchange of these nucleosomes likely enhances chromatin stability before nucleosomes are evicted during a new round of transcription. H3K36me3 is placed by SET2-related enzymes following active transcription and plays a role in facilitating nucleosome deacetylation [37,47]. Histone deacetylation stabilizes nucleosomes and thus may result in the observed negative correlation between H3K36me3 level and H3.3 turnover in gene body regions.

Our analyses suggest that heterochromatic regions turn over more slowly than euchromatic regions. We observed very slow turnover at telomeres and no turnover at all at pericentromeric regions, both of which are known to be enriched in H3.3. This suggests that pericentromeric nucleosomes are replaced only during replication, whereas telomeres undergo continuous exchange of their nucleosomes. Continuous exchange of nucleosomes at telomeres may be necessary for telomeric maintenance, although bulk telomeric nucleosomes may be exchanged during replication.

In summary, we have provided the basis for our understanding of nucleosome dynamics in mammals by providing a detailed picture of genome-wide replacement of the histone variant H3.3. This study will provide the platform for further studies into the determinants and mechanisms of nucleosome exchange.

## Conclusion

In this study, we mapped the genome-wide H3.3-specific nucleosome occupancy and the dynamic turnover of this histone variant in mammalian cells. We found that H3.3 turnover rates vary dramatically across functionally distinct genomic regions: turnover was highest at promoters and enhancers, intermediate at gene bodies and slowest at telomeres. Furthermore, we delineated striking correlations between turnover rates, histone modifications and H2A.Z, suggesting that intrinsic nucleosome properties such as histone modifications and histone variant inclusions are important properties of nucleosome stability.

## Materials and methods

### Cell line derivation

cDNA of human H3.3B or mouse H3.1 (Hist1h3h) was subcloned in-frame with a HA and FLAG tag at the carboxyl terminus and cloned into the lentiviral plvx-Tight-Puro Vector (Clontech, Mountain View, CA, USA). Lentiviral particles were packaged in 293 T cells with the psPAX2 packaging plasmid. Subsequently, we transduced NIH/3 T3 Tet-On® 3G cells (Clontech) and drug-selected with puromycin for stable integration.

### Western blotting

Cells were lysed with RIPA buffer, and whole-cell lysates were resolved on SDS-PAGE, transferred onto nitrocellulose membranes, and blotted with anti-HA (Roche, Basel, Switzerland), anti-FLAG (M2, Sigma, St. Louis, MO, USA) antibodies at 1:1,000. For anti-H3.3 western blotting, histones were isolated by acid extraction as described in [48] prior to immunoblotting with an anti-H3.3 antibody (Abcam, ab62642, Cambridge, UK). The anti-H3.3 antibody recognizes serine 31 of H3.3, which is not present on either H3.1 or H3.2, but cross-reactivity with other histone variants has not been tested experimentally (Abcam).

### Cell cycle analysis

Cells were examined for BrdU incorporation with the BrdU Labeling and Detection kit (Roche). For further cell cycle and DNA content analysis, cells were fixed with 70% ethanol, and subsequently stained with 50 μg/ml propidium iodide in 0.1% PBS-Triton-X with 0.2 mg/ml RNase A before analysis by flow cytometry.

### Time course induction and preparation of ChIP-Seq/RNA-Seq libraries

NIH/3 T3 MEFs were cultured in standard conditions with medium containing TET-compatible fetal bovine serum. MEFs were grown to 100% confluence and treated with 1 μg/ml aphidicolin for 18 hours. Adding 3 μg/ml doxycycline hyclate before crosslinking with formaldehyde at various time points induced HA/FLAG-H3.3 expression. ChIP-Seq experiments were performed as described previously [49] with an antibody against HA. In order to correlate HA/FLAG-H3.3 turnover with the presence of histone modifications and/or gene expression levels, we prepared ChIP-Seq and RNA-Seq from cells that were treated with aphidicolin for 18 hours prior to crosslinking and cell lysis.

### Data analysis

#### ChIP-Seq read mapping and peak calling

ChIP-Seq reads were mapped to the mouse genome (mm9, or NCBI 37) using Bowtie [50], only allowing ‘one read per position’ matching. Redundant reads were removed from each ChIP-Seq library. We identified ChIP-Seq tag-enriched peaks using SICER [51], which takes advantage of the enrichment information from neighboring bins to identify spatial clusters of signals that are unlikely to appear by chance. In this way, we could identify the dispersed epigenomic domains such as broad H3.3 domains on gene bodies. For calling of H3.3 peaks, we set the window size to 200 bp and gap size to 600 bp. Peaks with a false discovery rate higher than 0.01 were excluded from the analysis, compared with the input control libraries.

#### Analysis of H3.3 enrichment in genic regions and repetitive elements

The RefSeq gene annotation (mm9 or NCBI37) was downloaded from the UCSC browser. The mouse genome was classified into 5’ UTR, exon, intron, 3’ UTR and intergenic based on the UCSC annotation. Then we defined another two categories: promoter (3 kb upstream of the TSS) and TES (from TES to 5 kb downstream). The total mapped reads in each category were normalized to its total length. All mouse consensus repetitive element sequences, including pericentromeric element, L1, SINE1 and telomeric element, were obtained from Repbase Update (RU) [52]. The relative enrichment of H3.3 in each category of repetitive element was calculated by the proportions of reads mapped to repetitive elements normalized over input.

#### H3.3 peak annotation and distribution profiling

We used the position of the center of each peak for peak annotation. Two peaks from different libraries were considered as overlapping if the overlapped region accounts for 10% of the length of one peak. We classified genes with RPKM (reads per kilobase of exon model per million reads) <1 as lowest (inactive) genes and the other genes as active genes. We grouped genes into five categories with equal numbers of genes (highest, high, intermediate, low, lowest) based on their RPKM values. For each gene, reads falling in peaks were counted according to their shifted positions in 50 bp windows for the regions from 3 kb upstream of the TSS to the TSS and from the TES to 5 kb downstream of the TES. Within gene bodies, reads falling in peaks were counted according to their shifted positions in windows equal to 1% length of each gene. The number of reads in each window was normalized by the total number of bases within the window, and the total number of peak-filtered reads in the corresponding sample to obtain a normalized read-tag density.

#### RNA-Seq data analysis

The reads from RNA-Seq libraries were mapped to the mouse genome (mm9, or NCBI37) using TopHat [53], a fast splice junction mapper. The gene expression level was measured by RPKM.

#### Quantifying peak dynamics

The total number of H3.3 peaks was identified by peak-calling at the 72-hour time point when both early appearing and late appearing peaks were readily detected. The number of reads in each of these peak regions was recorded and normalized over the total mapped reads for each ChIP-Seq library. The relative H3.3 enrichment of each peak was calculated by normalizing the normalized reads in the peak over the normalized reads in input. A linear regression model was used to calculate turnover indices (*TI*) for each individual peak. Assuming that enrichment of H3.3 at 0 hour is E_0_, then E_t_ = *TI* × *t* + E_0_, where E_t_ equals H3.3 enrichment at each time point (E_1_, E_2_, E_3_,…E_n_), t = time point (1, 2, 3,…n). For peaks that reached their maximum enrichment before the end time point of analysis (*t* = 72 h), multiple linear regression coefficients were calculated by fitting the end time points from time point of maximum enrichment to 72 h and *t* was adjusted correspondingly. We adopted the regression coefficient with the best fit (smallest *P*-value) as the turnover index of the peaks. The turnover index was scaled from 0 and 1 in order to compare the reproducibility between duplicate experiments.

### Accession numbers

Our ChIP-seq and RNA-seq data sets have been deposited in the Gene Expression Omnibus data base with accession number GSE51505.

## Abbreviations

BP: Base pair; ChIP: Chromatin immunoprecipitation; DOX: Doxycycline; ESC: Embryonic stem cell; HA: Hemagglutinin; MEF: Mouse embryonic fibroblast; Pol: Polymerase; RPKM: Reads per kilobase of exon model per million reads; TES: Transcription end site; TET: Tetracycline; TSS: Transcription start site; UTR: Untranslated region.

## Competing interests

The authors declare that they have no competing interests.

## Authors’ contributions

DK participated in the design of the study, carried out experiments and drafted the manuscript. WJ carried out the data analysis and drafted the manuscript. AM participated in the construct generation. AB participated in the data analysis. MH participated in the data analysis. KZ conceived of the study, and participated in its design and coordination and helped to draft the manuscript. All authors read and approved the final manuscript.

## Supplementary Material

Additional file 1: Figure S1 Fast turnover of enhancer H3.3 nucleosomes are associated with higher active histone modification and lower repressive modification. The H3.3-containing nucleosomes at enhancers were separated into two groups (fast turnover and slow turnover) based on the distribution of turnover indices in Figure 4G. The histone modification levels of the two groups are displayed and compared using box plots. Click here for file

## References

[B1] Jin C Zang C Wei G Cui K Peng W Zhao K Felsenfeld G H3.3/H2A.Z double variant-containing nucleosomes mark 'nucleosome-free regions' of active promoters and other regulatory regions Nat Genet 2009 14 941 945 10.1038/ng.409 19633671 PMC3125718

[B2] Jin C Felsenfeld G Nucleosome stability mediated by histone variants H3.3 and H2A.Z Genes Dev 2007 14 1519 1529 10.1101/gad.1547707 17575053 PMC1891429

[B3] Ng RK Gurdon JB Epigenetic memory of an active gene state depends on histone H3.3 incorporation into chromatin in the absence of transcription Nat Cell Biol 2008 14 102 109 10.1038/ncb1674 18066050

[B4] Hu G Cui K Northrup D Liu C Wang C Tang Q Ge K Levens D Crane-Robinson C Zhao K H2A.Z facilitates access of active and repressive complexes to chromatin in embryonic stem cell self-renewal and differentiation Cell Stem Cell 2013 14 180 192 10.1016/j.stem.2012.11.003 23260488 PMC3570599

[B5] Henikoff S Furuyama T Ahmad K Histone variants, nucleosome assembly and epigenetic inheritance Trends Genet 2004 14 320 326 10.1016/j.tig.2004.05.004 15219397

[B6] Elsaesser SJ Goldberg AD Allis CD New functions for an old variant: no substitute for histone H3.3 Curr Opin Genet Dev 2010 14 110 117 10.1016/j.gde.2010.01.003 20153629 PMC2860041

[B7] Ahmad K Henikoff S The histone variant H3.3 marks active chromatin by replication-independent nucleosome assembly Mol Cell 2002 14 1191 1200 10.1016/S1097-2765(02)00542-7 12086617

[B8] Ray-Gallet D Quivy JP Scamps C Martini EM Lipinski M Almouzni G HIRA is critical for a nucleosome assembly pathway independent of DNA synthesis Mol Cell 2002 14 1091 1100 10.1016/S1097-2765(02)00526-9 12049744

[B9] Goldberg AD Banaszynski LA Noh KM Lewis PW Elsaesser SJ Stadler S Dewell S Law M Guo X Li X Wen D Chapgier A DeKelver RC Miller JC Lee YL Boydston EA Holmes MC Gregory PD Greally JM Rafii S Yang C Scambler PJ Garrick D Gibbons RJ Higgs DR Cristea IM Urnov FD Zheng D Allis CD Distinct factors control histone variant H3.3 localization at specific genomic regions Cell 2010 14 678 691 10.1016/j.cell.2010.01.003 20211137 PMC2885838

[B10] Wong LH McGhie JD Sim M Anderson MA Ahn S Hannan RD George AJ Morgan KA Mann JR Choo KH ATRX interacts with H3.3 in maintaining telomere structural integrity in pluripotent embryonic stem cells Genome Res 2010 14 351 360 10.1101/gr.101477.109 20110566 PMC2840985

[B11] Lewis PW Elsaesser SJ Noh KM Stadler SC Allis CD Daxx is an H3.3-specific histone chaperone and cooperates with ATRX in replication-independent chromatin assembly at telomeres Proc Natl Acad Sci U S A 2010 14 14075 14080 10.1073/pnas.1008850107 20651253 PMC2922592

[B12] Szenker E Lacoste N Almouzni G A developmental requirement for HIRA-dependent H3.3 deposition revealed at gastrulation in Xenopus Cell Rep 2012 14 730 740 10.1016/j.celrep.2012.05.006 22813747

[B13] Hake SB Garcia BA Duncan EM Kauer M Dellaire G Shabanowitz J Bazett-Jones DP Allis CD Hunt DF Expression patterns and post-translational modifications associated with mammalian histone H3 variants J Biol Chem 2006 14 559 568 10.1074/jbc.M509266200 16267050

[B14] Schwartz BE Ahmad K Transcriptional activation triggers deposition and removal of the histone variant H3.3 Genes Dev 2005 14 804 814 10.1101/gad.1259805 15774717 PMC1074318

[B15] Mito Y Henikoff JG Henikoff S Genome-scale profiling of histone H3.3 replacement patterns Nat Genet 2005 14 1090 1097 10.1038/ng1637 16155569

[B16] Tamura T Smith M Kanno T Dasenbrock H Nishiyama A Ozato K Inducible deposition of the histone variant H3.3 in interferon-stimulated genes J Biol Chem 2009 14 12217 12225 10.1074/jbc.M805651200 19244243 PMC2673290

[B17] Stroud H Otero S Desvoyes B Ramirez-Parra E Jacobsen SE Gutierrez C Genome-wide analysis of histone H3.1 and H3.3 variants in Arabidopsis thaliana Proc Natl Acad Sci U S A 2012 14 5370 5375 10.1073/pnas.1203145109 22431625 PMC3325649

[B18] Bai L Morozov AV Gene regulation by nucleosome positioning Trends Genet 2010 14 476 483 10.1016/j.tig.2010.08.003 20832136

[B19] Svaren J Horz W Transcription factors vs nucleosomes: regulation of the PHO5 promoter in yeast Trends Biochem Sci 1997 14 93 97 10.1016/S0968-0004(97)01001-3 9066259

[B20] Schones DE Cui K Cuddapah S Roh TY Barski A Wang Z Wei G Zhao K Dynamic regulation of nucleosome positioning in the human genome Cell 2008 14 887 898 10.1016/j.cell.2008.02.022 18329373 PMC10894452

[B21] Mulholland N Xu Y Sugiyama H Zhao K SWI/SNF-mediated chromatin remodeling induces Z-DNA formation on a nucleosome Cell Biosci 2012 14 3 10.1186/2045-3701-2-3 22264354 PMC3293710

[B22] Schnitzler G Sif S Kingston RE Human SWI/SNF interconverts a nucleosome between its base state and a stable remodeled state Cell 1998 14 17 27 10.1016/S0092-8674(00)81217-9 9674423

[B23] Boeger H Griesenbeck J Strattan JS Kornberg RD Removal of promoter nucleosomes by disassembly rather than sliding in vivo Mol Cell 2004 14 667 673 10.1016/j.molcel.2004.05.013 15175161

[B24] Struhl K Segal E Determinants of nucleosome positioning Nat Struct Mol Biol 2013 14 267 273 10.1038/nsmb.2506 23463311 PMC3740156

[B25] Giresi PG Gupta M Lieb JD Regulation of nucleosome stability as a mediator of chromatin function Curr Opin Genet Dev 2006 14 171 176 10.1016/j.gde.2006.02.003 16503136

[B26] Dion MF Kaplan T Kim M Buratowski S Friedman N Rando OJ Dynamics of replication-independent histone turnover in budding yeast Science 2007 14 1405 1408 10.1126/science.1134053 17347438

[B27] Jamai A Imoberdorf RM Strubin M Continuous histone H2B and transcription-dependent histone H3 exchange in yeast cells outside of replication Mol Cell 2007 14 345 355 10.1016/j.molcel.2007.01.019 17289583

[B28] Rufiange A Jacques PE Bhat W Robert F Nourani A Genome-wide replication-independent histone H3 exchange occurs predominantly at promoters and implicates H3 K56 acetylation and Asf1 Mol Cell 2007 14 393 405 10.1016/j.molcel.2007.07.011 17679090

[B29] Deal RB Henikoff S Capturing the dynamic epigenome Genome Biol 2010 14 218 10.1186/gb-2010-11-10-218 20959022 PMC3218653

[B30] Deal RB Henikoff JG Henikoff S Genome-wide kinetics of nucleosome turnover determined by metabolic labeling of histones Science 2010 14 1161 1164 10.1126/science.1186777 20508129 PMC2879085

[B31] Jansen LE Black BE Foltz DR Cleveland DW Propagation of centromeric chromatin requires exit from mitosis J Cell Biol 2007 14 795 805 10.1083/jcb.200701066 17339380 PMC2064054

[B32] Ray-Gallet D Woolfe A Vassias I Pellentz C Lacoste N Puri A Schultz DC Pchelintsev NA Adams PD Jansen LE Almouzni G Dynamics of histone H3 deposition in vivo reveal a nucleosome gap-filling mechanism for H3.3 to maintain chromatin integrity Mol Cell 2011 14 928 941 10.1016/j.molcel.2011.12.006 22195966

[B33] Kimura H Cook PR Kinetics of core histones in living human cells: little exchange of H3 and H4 and some rapid exchange of H2B J Cell Biol 2001 14 1341 1353 10.1083/jcb.153.7.1341 11425866 PMC2150718

[B34] Meshorer E Yellajoshula D George E Scambler PJ Brown DT Misteli T Hyperdynamic plasticity of chromatin proteins in pluripotent embryonic stem cells Dev Cell 2006 14 105 116 10.1016/j.devcel.2005.10.017 16399082 PMC1868458

[B35] Creyghton MP Cheng AW Welstead GG Kooistra T Carey BW Steine EJ Hanna J Lodato MA Frampton GM Sharp PA Boyer LA Young RA Jaenisch R Histone H3K27ac separates active from poised enhancers and predicts developmental state Proc Natl Acad Sci U S A 2010 14 21931 21936 10.1073/pnas.1016071107 21106759 PMC3003124

[B36] Rada-Iglesias A Bajpai R Swigut T Brugmann SA Flynn RA Wysocka J A unique chromatin signature uncovers early developmental enhancers in humans Nature 2011 14 279 283 10.1038/nature09692 21160473 PMC4445674

[B37] Li B Jackson J Simon MD Fleharty B Gogol M Seidel C Workman JL Shilatifard A Histone H3 lysine 36 dimethylation (H3K36me2) is sufficient to recruit the Rpd3s histone deacetylase complex and to repress spurious transcription J Biol Chem 2009 14 7970 7976 10.1074/jbc.M808220200 19155214 PMC2658090

[B38] Carrozza MJ Li B Florens L Suganuma T Swanson SK Lee KK Shia WJ Anderson S Yates J Washburn MP Workman JL Histone H3 methylation by Set2 directs deacetylation of coding regions by Rpd3S to suppress spurious intragenic transcription Cell 2005 14 581 592 10.1016/j.cell.2005.10.023 16286007

[B39] Barski A Chepelev I Liko D Cuddapah S Fleming AB Birch J Cui K White RJ Zhao K Pol II and its associated epigenetic marks are present at Pol III-transcribed noncoding RNA genes Nat Struct Mol Biol 2010 14 629 634 10.1038/nsmb.1806 20418881 PMC2917008

[B40] Cacchione S Cerone MA Savino M In vitro low propensity to form nucleosomes of four telomeric sequences FEBS Lett 1997 14 37 41 10.1016/S0014-5793(96)01318-X 9000509

[B41] Adkins MW Tyler JK Transcriptional activators are dispensable for transcription in the absence of Spt6-mediated chromatin reassembly of promoter regions Mol Cell 2006 14 405 416 10.1016/j.molcel.2005.12.010 16455495

[B42] Zaret KS Caravaca JM Tulin A Sekiya T Nuclear mobility and mitotic chromosome binding: similarities between pioneer transcription factor FoxA and linker histone H1 Cold Spring Harb Symp Quant Biol 2010 14 219 226 10.1101/sqb.2010.75.061 21502411

[B43] Ballare C Castellano G Gaveglia L Althammer S Gonzalez-Vallinas J Eyras E Le Dily F Zaurin R Soronellas D Vicent GP Beato M Nucleosome-driven transcription factor binding and gene regulation Mol Cell 2013 14 67 79 23177737 10.1016/j.molcel.2012.10.019

[B44] Schwabish MA Struhl K The Swi/Snf complex is important for histone eviction during transcriptional activation and RNA polymerase II elongation in vivo Mol Cell Biol 2007 14 6987 6995 10.1128/MCB.00717-07 17709398 PMC2168902

[B45] Teves SS Henikoff S Heat shock reduces stalled RNA polymerase II and nucleosome turnover genome-wide Genes Dev 2011 14 2387 2397 10.1101/gad.177675.111 22085965 PMC3222904

[B46] Kaplan CD Laprade L Winston F Transcription elongation factors repress transcription initiation from cryptic sites Science 2003 14 1096 1099 10.1126/science.1087374 12934008

[B47] Kizer KO Phatnani HP Shibata Y Hall H Greenleaf AL Strahl BD A novel domain in Set2 mediates RNA polymerase II interaction and couples histone H3 K36 methylation with transcript elongation Mol Cell Biol 2005 14 3305 3316 10.1128/MCB.25.8.3305-3316.2005 15798214 PMC1069628

[B48] Shechter D Dormann HL Allis CD Hake SB Extraction, purification and analysis of histones Nat Protoc 2007 14 1445 1457 10.1038/nprot.2007.202 17545981

[B49] Barski A Cuddapah S Cui K Roh TY Schones DE Wang Z Wei G Chepelev I Zhao K High-resolution profiling of histone methylations in the human genome Cell 2007 14 823 837 10.1016/j.cell.2007.05.009 17512414

[B50] Langmead B Trapnell C Pop M Salzberg SL Ultrafast and memory-efficient alignment of short DNA sequences to the human genome Genome Biol 2009 14 R25 10.1186/gb-2009-10-3-r25 19261174 PMC2690996

[B51] Zang C Schones DE Zeng C Cui K Zhao K Peng W A clustering approach for identification of enriched domains from histone modification ChIP-Seq data Bioinformatics 2009 14 1952 1958 10.1093/bioinformatics/btp340 19505939 PMC2732366

[B52] Jurka J Kapitonov VV Pavlicek A Klonowski P Kohany O Walichiewicz J Repbase Update, a database of eukaryotic repetitive elements Cytogenet Genome Res 2005 14 462 467 10.1159/000084979 16093699

[B53] Kim D Pertea G Trapnell C Pimentel H Kelley R Salzberg SL TopHat2: accurate alignment of transcriptomes in the presence of insertions, deletions and gene fusions Genome Biol 2013 14 R36 10.1186/gb-2013-14-4-r36 23618408 PMC4053844

