# Erratum to: Genome-wide incorporation dynamics reveal distinct categories of turnover for the histone variant H3.3

**DOI:** 10.1186/s13059-016-0886-3

**Published:** 2016-02-04

**Authors:** Daniel C. Kraushaar, Wenfei Jin, Alika Maunakea, Brian Abraham, Misook Ha, Keji Zhao

**Affiliations:** 1 Systems Biology Center, National Heart, Lung, and Blood Institute, NIH, Bethesda, MD 20892 USA; 2 Samsung Advanced Institute of Technology, Samsung Electronics Corporation, Yongin-Si, Gyeonggi-Do 446-712 South Korea

After the publication of this work [1] an error was noticed in Fig. 1d. In the DAPI columns the same image was used accidentally for the 48 h and 72 h timepoints. The corrected figure is shown below. We apologize for this error.

**Fig. 1:**
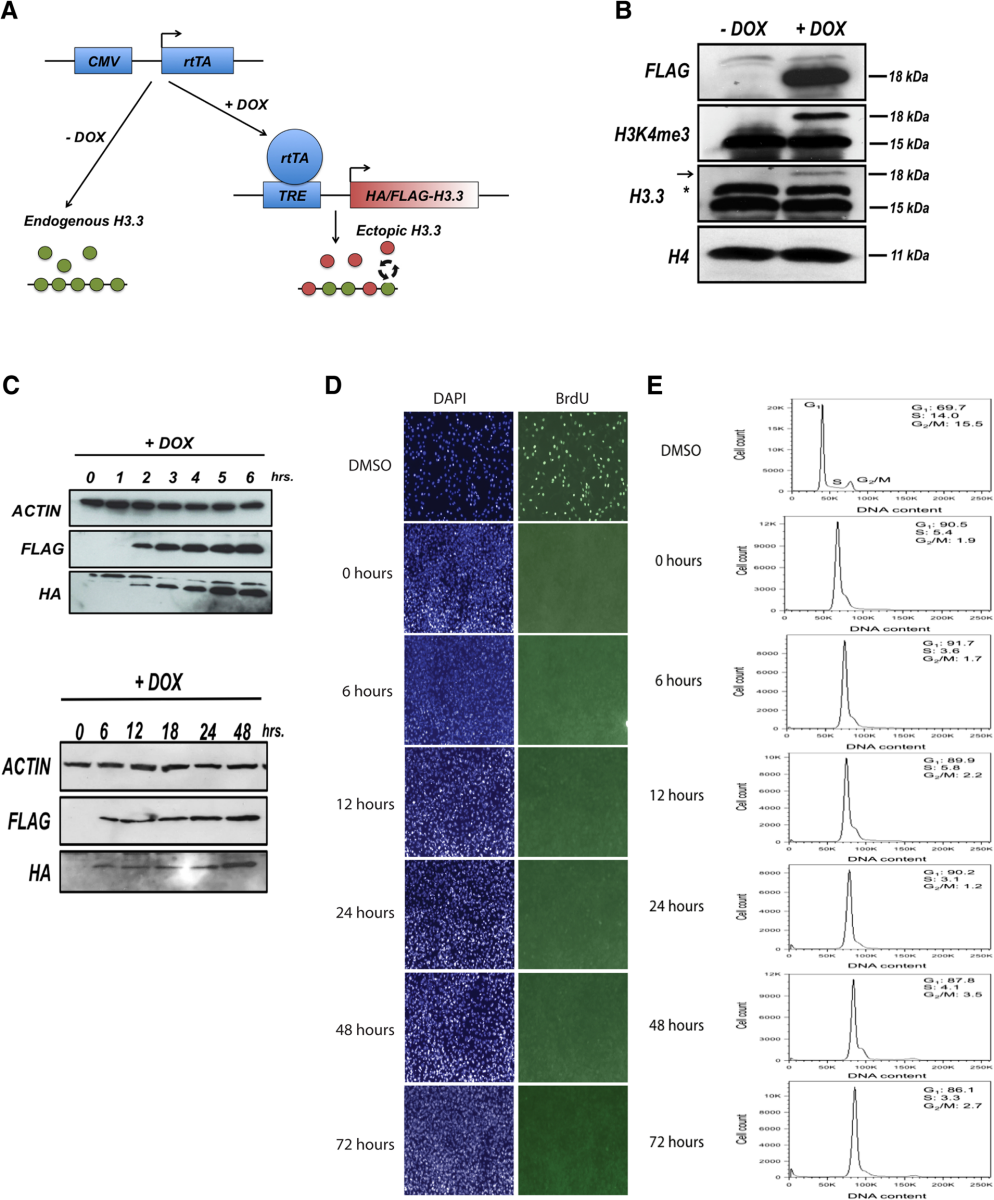
A versatile system to study replication-independent nucleosome dynamics in mammals. (**a**) Schematic of TET-inducible expression system to study H3.3 turnover. CMV, cytomegalovirus; rtTA, reverse tetracycline-controlled transactivator; TRE, tetracycline responsive elements. (**b**) Western blot showing protein levels of transgenic HA/FLAG-H3.3 compared to endogenous H3.3. HA/FLAG-H3.3 expression 24 hours after DOX addition. The band marked with an asterisk is non-specific. The arrow marks transgenic HA/FLAG-H3.3. (**c**) Time course western blots of HA/FLAG-H3.3 expression. (**d**) Bromodeoxyuridine (BrdU) immunostaining of NIH/3 T3 cells treated with DNA polymerase inhibitor aphidicolin and DOX across time points of H3.3 induction. DMSO, dimethylsulfoxide. (**e**) Cell cycle analysis of cells treated with aphidicolin/DOX. Cells were stained with propidium iodide and analyzed by flow cytometry
