# Supplementary figures and images for: Genome-wide incorporation dynamics reveal distinct categories of turnover for the histone variant H3.3
Source: Genome Biol. 2013 Oct 31;14(10):R121. doi: 10.1186/gb-2013-14-10-r121 (PMC3983652; doi:10.1186/gb-2013-14-10-r121)

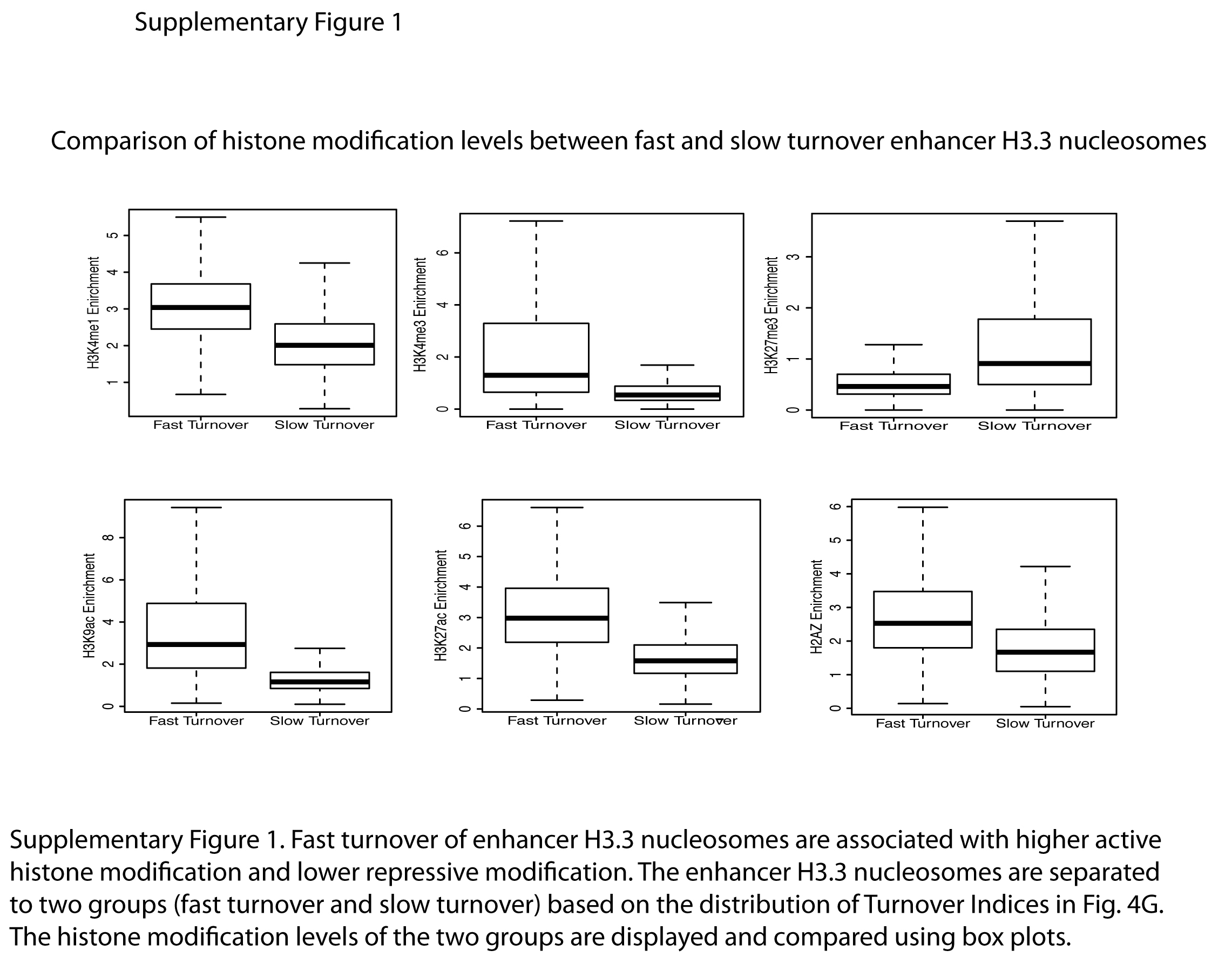

Supplement: Additional file 1: Figure S1 — Fast turnover of enhancer H3.3 nucleosomes are associated with higher active histone modification and lower repressive modification. The H3.3-containing nucleosomes at enhancers were separated into two groups (fast turnover and slow turnover) based on the distribution of turnover indices in Figure 4G. The histone modification levels of the two groups are displayed and compared using box plots. [file gb-2013-14-10-r121-S1.jpeg]
